# Indole-3-Acetic Acid Biosynthesis Pathways in the Plant-Beneficial Bacterium *Arthrobacter pascens* ZZ21

**DOI:** 10.3390/ijms19020443

**Published:** 2018-02-01

**Authors:** Mengsha Li, Rui Guo, Fei Yu, Xu Chen, Haiyan Zhao, Huixin Li, Jun Wu

**Affiliations:** 1 Soil Ecology Lab, College of Resources and Environmental Science, Nanjing Agricultural University, Nanjing 210095, China; 2014203018@njau.edu.cn (M.L.); 2015203007@njau.edu.cn (R.G.); 2014203007@njau.edu.cn (F.Y.); 2015203006@njau.edu.cn (X.C.); 2 College of Resources and Environmental Science, Nanjing Agricultural University, Nanjing 210095, China; haiyanzhao@njau.edu.cn

**Keywords:** *Arthrobacter*, IAA biosynthesis pathway, genome-wide analysis, metabolomics, HPLC-MS

## Abstract

*Arthrobacter pascens* ZZ21 is a plant-beneficial, fluoranthene-degrading bacterial strain found in the rhizosphere. The production of the phytohormone indole-3-aectic acid (IAA) by ZZ21 is thought to contribute to its ability to promote plant growth and remediate fluoranthene-contaminated soil. Using genome-wide analysis combined with metabolomic and high-performance liquid chromatography-mass spectrometry (HPLC-MS) analyses, we characterized the potential IAA biosynthesis pathways in *A. pascens* ZZ21. IAA production increased 4.5-fold in the presence of 200 mg·L^−1^ tryptophan in the culture medium. The transcript levels of *prr* and *aldH*, genes which were predicted to encode aldehyde dehydrogenases, were significantly upregulated in response to exogenous tryptophan. Additionally, metabolomic analysis identified the intermediates indole-3-acetamide (IAM), indole-3-pyruvic acid (IPyA), and the enzymatic reduction product of the latter, indole-3-lactic acid (ILA), among the metabolites of ZZ21, and subsequently also IAM, ILA, and indole-3-ethanol (TOL), which is the enzymatic reduction product of indole-3-acetaldehyde, by HPLC-MS. These results suggest that the tryptophan-dependent IAM and IPyA pathways function in ZZ21.

## 1. Introduction

Indole-3-acetic acid (IAA), the most common naturally occurring auxin, is a hormone produced by plants, fungi and bacteria. IAA plays a central role in modulating plant growth and development [1,2,3]. In addition to being produced by plants, IAA is produced by some beneficial bacteria in the rhizosphere, where it acts as a signaling molecule that has significant effects on the communication between plants and microorganisms and promotes plant growth [4,5,6]. Tryptophan (Trp) is a main precursor for IAA biosynthesis in microbes [5]. Based on the distinct intermediates involved in tryptophan-dependent IAA biosynthesis, five different pathways have been characterized in bacteria: the indole-3-acetamide (IAM), indole-3-pyruvic acid (IPyA), indole-3-acetonitrile (IAN), tryptamine (TAM), and tryptophan side-chain oxidase (TSO) pathways [7,8]. Although the tryptophan-independent pathway is also thought to occur in bacteria [9], no specific enzymes in this pathway have been characterized.

Pathways and key genes involved in IAA biosynthesis in Gram-negative bacteria are well documented, but reports on the biosynthesis pathways used by Gram-positive bacteria are lacking. The Gram-positive, spore-forming bacterium *Paenibacillus polymyxa* E681 was shown to synthesize IAA via the IPyA pathway, and the key gene *ipdC* (encoding indole-3-pyruvate decarboxylase) was functionally identified and found to be constitutively expressed [10]. The main biosynthetic route used by the phytopathogen *Rhodococcus fascians* is the IPyA pathway [11]. The IAN, TAM and IPyA pathways and an uncharacterized pathway are thought to function in *Bacillus amyloliquefaciens* SQR9. The genes *patB* (encoding a conserved hypothetical protein predicted to be an aminotransferase), *yclC* (encoding a UbiD family decarboxylase) and *dhaS* (encoding indole 3-acetaldehyde dehydrogenase) constitute a putative complete IPyA pathway that mediates the biosynthesis of IAA in this bacterium [12]. *Arthrobacter*, one of the largest groups of soil bacteria, produces IAA when tryptophan is present in the medium [13,14,15,16]. An early study by Mino demonstrated that the IPyA and IAM pathways function in *Arthrobacter* spp. [17]. However, the details of IAA biosynthesis that function in *Arthrobacter* still require further study.

*Arthrobacter pascens* ZZ21 was isolated from the rhizosphere of forest soil from Zijin Mountain in Nanjing City, Jiangsu Province, China. It is a multifunctional, salt-tolerant bacterial strain that secretes IAA, dissolves inorganic phosphorus and degrades fluoranthene, which belongs to the polycyclic aromatic hydrocarbons (PAHs) class of persistent organic pollutants (POPs). IAA production in *A. pascens* ZZ21 can reach a level of 92.31 mg·L^−1^ under optimal conditions (pH 6, liquid volume of 45 mL medium in a 150 mL conical flask, temperature of 28 °C, shaking time of 15 h, and mannitol and yeast extract as carbon and nitrogen sources respectively) [18]. We previously reported that the IAA produced by *A. pascens* ZZ21 increases soil microbial activities and contributes to its plant-growth-promoting effects. We also demonstrated that the application of IAA-producing *A. pascens* ZZ21 combined with the fluoranthene-degrading strain *Bacillus cereus* Z21 is more effective at remediating fluoranthene-contaminated soil than ZZ21 alone [19]. IAA produced by *A. pascens* ZZ21 promotes plant growth and enhances tolerance to fluoranthene, leading to increased fluoranthene uptake by plants, thereby improving a plant’s ability to remediate fluoranthene-contaminated soils.

In the current study, using a combination of genetic and chemical analyses, we characterized potential IAA biosynthesis pathways in *A. pascens* ZZ21. We identified candidate genes involved in IAA biosynthesis through genome-wide screening and examined their transcriptional responses to exogenous tryptophan. Finally, we detected potential intermediates involved in possible IAA biosynthesis pathways using metabolomic analysis and HPLC-MS, thereby shedding light on the regulatory mechanism underlying the soil-remediation and plant-growth-promoting effects of this bacterium.

## 2. Results

### 2.1. Whole-Genome Sequencing of A. pascens ZZ21 and Screening for Genes Likely Involved in IAA Biosynthesis

We assembled the entire *A. pascens* ZZ21 genome and mined it for genes involved in IAA metabolism. Sequencing data statistics are listed in Table S1. Statistical parameters used for the genome assembly are indicated in Table S2, and gene information statistics are listed in Table S3. Based on the reported genes and IAA biosynthesis pathways [4,8,20], we performed a Blast analysis of the predicted ZZ21 genes against the Kyoto Encyclopedia of Genes and Genomes (KEGG) and National Center for Biotechnology Information (NCBI) databases using their deduced protein sequences as queries and identified candidate genes involved in tryptophan-dependent IAA biosynthesis (Figure S1; Table 1). For the IAM pathway, comparative sequence analyses showed that the deduced protein sequence of *iaaM* (orf0652) in ZZ21 had obviously sequence similarity to characterized tryptophan 2-monoxygenase, sharing at 88% identification with tryptophan 2-monxygenase from *Arthrobacter* sp. P2b (GenBank accession No. SLJ94339.1) and 89% identification with tryptophan 2-monxygenase from *Arthrobacter* sp. ok362 (GenBank accession No. SDK38081.1). The deduced protein sequences of genes *aam* (orf3469) and *gatA* (orf0389) shared 90% identification with amidase from *Arthrobacter* sp. Soil736 (NCBI Reference Sequence No. WP_056629692.1) and 81% identification with amidase from *Arthrobacter globiformis* (NCBI Reference Sequence No. WP_087872787.1), respectively. We identified the *iaaM*-homologous gene, which was predicted to encode tryptophan 2-monooxygenase, and the amidase genes (*aam*, *gatA*), which may function in the IAM hydrolysis reaction (Table 1). The final step in the IPyA, TAM, and TSO pathways is the oxidative dehydrogenation of indole-3-acetaldehyde (IAAld) to IAA, which is catalyzed by aldehyde dehydrogenase. Comparative sequence analyses showed that the deduced protein sequences of *prr* (orf1423), *puuC* (orf2422) and *aldH* (orf3473) had an obvious overall sequence similarity to characterized aldehyde dehydrogenase, respectively sharing 96% identification with aldehyde dehydrogenase from *Arthrobacter nitrophenolicus* (GenBank accession No. ELT45240.1), 94% identification with aldehyde dehydrogenase from *Arthrobacter globiformis* (NCBI Reference Sequence No. WP_003803492.1) and 93% identification with aldehyde dehydrogenase from *Arthrobacter* sp. 35W (NCBI Reference Sequence No. WP_026555119.1). Thus, *prr*, *puuC*, and *aldH* might encode this enzyme in ZZ21. However, the putative amino transferase gene and the well-documented indole-3-pyruvate decarboxylase *ipdC* gene in the IPyA pathway, as well as genes encoding the putative amine oxidase and tryptophan decarboxylase in the TAM pathway, were not detected in the ZZ21 genome. Finally, no genes involved in the IAN pathway were found in the ZZ21 genome.

### 2.2. Identification of Candidate Genes Involved in Tryptophan-Dependent IAA Biosynthesis in A. pascens ZZ21 Based on Their Transcriptional Responses to Tryptophan

**Figure 1:**
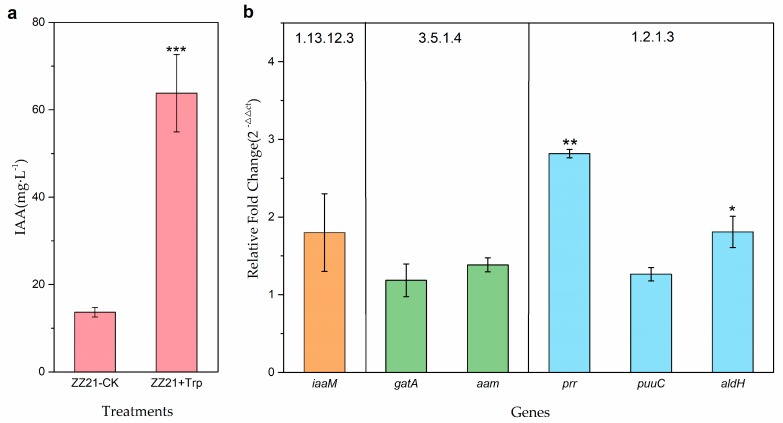
IAA production and genes transcript levels in ZZ21 in response to tryptophan. (**a**) IAA production in *A. pascens* ZZ21 cultured for 48 h with or without 200 mg·L^−1^ l-tryptophan in Luria-Bertani (LB) medium. (**b**) Relative expression of genes in ZZ21 cultured in the absence or presence of tryptophan (Trp). Gene transcript levels were evaluated by qRT-PCR. ZZ21 was grown in LB medium with or without tryptophan for 48 h. The 16sRNA gene of ZZ21 was used as an internal reference. Error bars represent standard errors of three biological replicates. One-way analysis of variance (ANOVA) was performed. * *p* < 0.05, ** *p* < 0.01, *** *p* < 0.001.

Since the candidate genes identified by genome-wide sequencing are likely involved in tryptophan-dependent IAA biosynthesis, we reviewed their transcriptional response to exogenous tryptophan in the culture medium. IAA production in ZZ21 increased 4.5-fold when 200 mg·L^−1^ l-tryptophan was added to the Luria-Bertani (LB) medium (Figure 1a), indicating that IAA biosynthesis in ZZ21 is significantly induced by tryptophan. Among the six candidate genes, only two were significantly induced by exogenous tryptophan (Figure 1b). The expression of *prr* and *aldH*, which were predicted to encode aldehyde dehydrogenases, increased 2.8-fold and 1.8-fold, respectively, in response to exogenous tryptophan. These two genes were annotated and predicted to participate in the final steps of the tryptophan-dependent IPyA, TAM and TSO IAA biosynthesis pathways. The expression of the four other candidate genes did not significantly change in response to exogenous tryptophan treatment, suggesting that these four genes were insensitive to exogenous tryptophan or did not actually function in IAA biosynthesis in ZZ21 (Figure 1b).

### 2.3. Detection of Intermediates in the IAA Biosynthesis Pathway in A. pascens ZZ21 by Metabolomic Analysis

We performed a metabolomic analysis to measure the levels of IAA biosynthesis intermediates in *A. pascens* ZZ21. We visualized the total ion current (TIC) of the *A. pascens* ZZ21 samples, which exhibited strong signals, a large peak capacity, and good reproducibility of retention times (Figure S2). Peak alignment of all raw data revealed 725 possible metabolites. After all internal standards and any known pseudo-positive peaks caused by background noise, column bleed, or the BSTFA (*N*, *O*-Bis(trimethylsilyl)trifluoroacetamide) derivatization procedure, were removed, a total of 213 metabolites were detected. 

**Figure 2:**
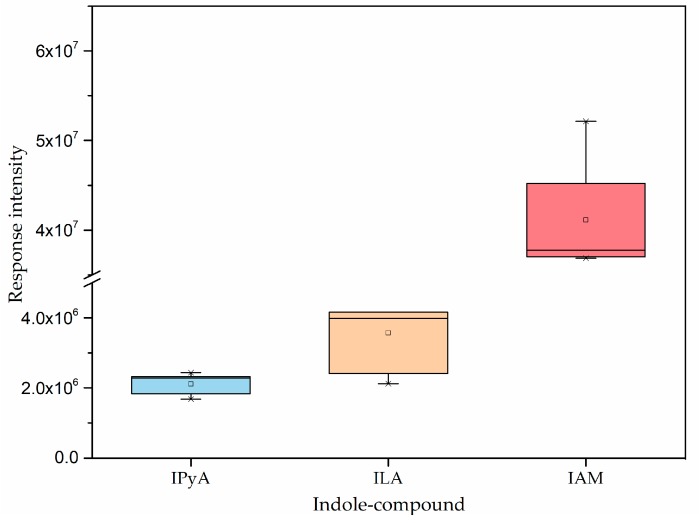
Indole derivatives detected among ZZ21 metabolites by metabolomic analysis. Bars represent standard deviations of five biological replicates. IPyA: indole-3-pyruvic acid; ILA: indole-3-lactic acid; IAM: indole-3-acetamide.

Based on the proposed IAA biosynthesis pathways in bacteria [4,8,20], we mined the metabolites of *A. pascens* ZZ21 for intermediates involved in each step of these pathways. Indole-3-pyruvic acid and indole-3-acetamide were detected among the metabolites of ZZ21 (Figure 2). The response intensity of IPyA was much lower than that of IAM, and IAAld was not detected among the metabolites of ZZ21, as IPyA and IAAld are unstable and accumulate at almost undetectable levels in culture [7,21]. Indole-3-lactic acid (ILA), the product of the enzymatic reduction of IPyA, was detected in ZZ21, suggesting that IAA biosynthesis in ZZ21 involves the IPyA pathway (Figure 2). 

### 2.4. Identification of Candidate Intermediates Involved in IAA Biosynthesis in A. pascens ZZ21 by HPLC-MS

**Figure 3:**
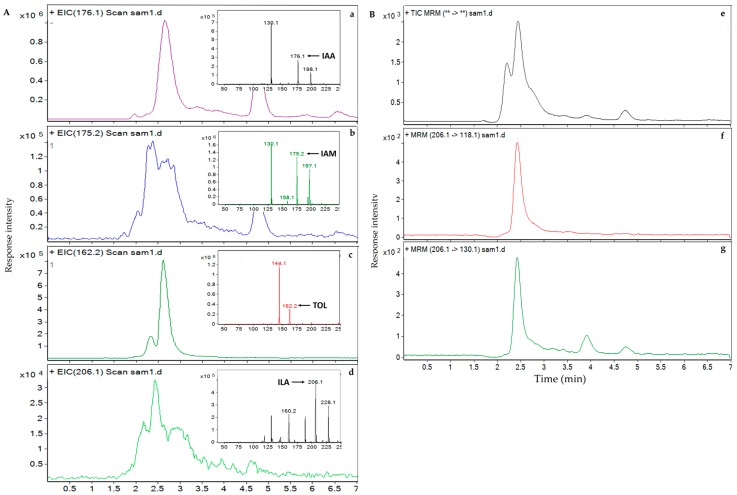
High-performance liquid chromatography–mass spectrometry (HPLC-MS) analysis of the four target indole compounds in ZZ21. (**A**) Extracted ion chromatography (EIC) of the four indole compounds involved in IAA biosynthesis in ZZ21: (**a**) IAA: indole-3-acetic acid, (**b**) IAM: indole-3-acetamide, (**c**) ILA: indole-3-lactic acid, and (**d**) TOL: indole-3-ethanol. The multiple reaction monitoring (MRM) chromatography of the four indole-compounds were showed on the top right part of each EIC figure. The fragmentor voltage was 135 V. (**B**) MRM chromatography of the characteristic fragments of indole-3-lactic acid in ZZ21 (**e**). 118.1 *m*/*z* (**f**) and 130.1 *m*/*z* (**g**) are two characteristic peaks of indole-3-lactic acid fragments. The fragmentor voltage was 90 V.

**Figure 4:**
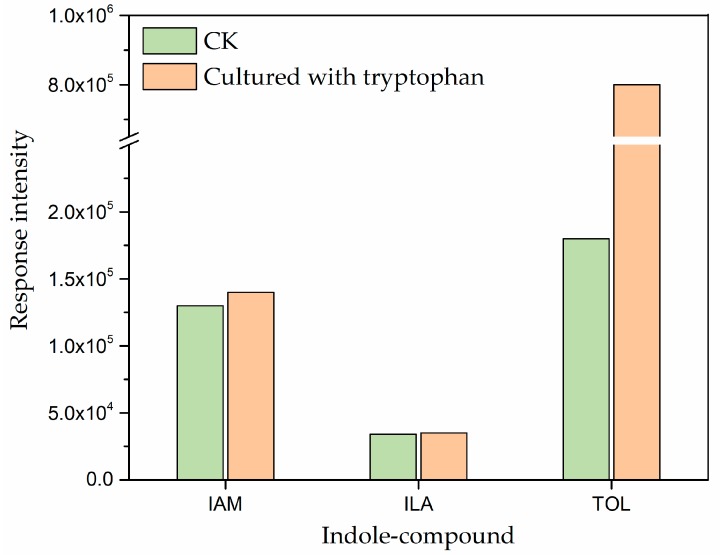
Analysis of the intermediates in ZZ21 grown in the medium with or without (CK: control check) tryptophan by HPLC-MS. IAM: indole-3-acetamide; ILA: indole-3-lactic acid; TOL: indole-3-ethanol.

Based on the results of the genome-wide sequencing and metabolomic analysis, we identified two possible IAA biosynthesis pathways in *A. pascens* ZZ21. To clarify the potential IAA biosynthesis pathway used by ZZ21, we further identified IAM (the key intermediate in the IAM pathway), ILA (the product of enzymatic reduction of IAAld in the IPyA pathway) and indole-3-ethanol (TOL) (an IAAld catabolic product) by HPLC-MS. The retention time of IAA, IAM, TOL and ILA in ZZ21 metabolites were similar to their standards. After hydrogenation, the relative molecular masses of the four indole compounds were 176.1, 175.2, 206.1, and 162.2, respectively (Figure 3A). IAM and TOL were detected in ZZ21 (Figure 3b,c), whereas no defined peak was detected for ILA (Figure 3d). Therefore, we used secondary ion mass spectrometry in an effort to identify ILA by detecting two characteristic ILA fragment peaks (Figure 3B). Two peaks at 118.1 (206.1 − 88 = C_8_H_7_N) *m*/*z* and 130.1 (206.1 − 88 = C_9_H_8_N) *m*/*z*, which are characteristic masses identified in ILA fragmentation patterns, were detected in ZZ21 metabolic samples (Figure 3f,g). The concentration of TOL was significantly increased when exogenous tryptophan was supplied to the medium (Figure 4). These results indicated that ILA is indeed present in ZZ21. Taken together, the IAM and IPyA pathways are thought to function in ZZ21.

## 3. Discussion

IAA biosynthesis in various *Arthrobacter* species (*A. crystallopoietes*, *A. globiformis*, *A. nicotianae*, *A. sulfonivorans*, and *A. sulfureus*) is stimulated by the application of exogenous tryptophan [13,14,15]. Similarly, in the current study, we detected a significant increase in IAA production after the addition of tryptophan to the ZZ21 culture medium, indicating that IAA biosynthesis in ZZ21 is induced by tryptophan (Figure 1a). To identify possible IAA biosynthesis pathways in ZZ21, we used three different methods involving combined genetic and chemical analyses.

**Figure 5:**
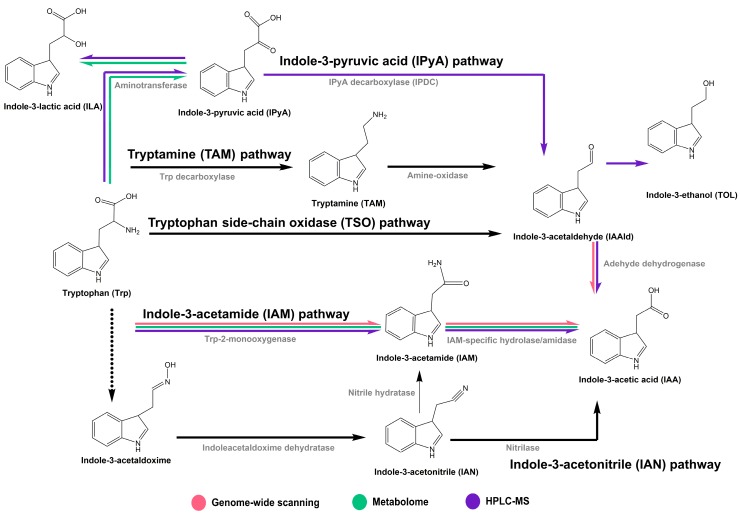
Tryptophan-dependent pathway for IAA biosynthesis in bacteria. IAM: indole-3-acetamide; IPyA: indole-3-pyruvic acid; IAAld: indole-3-acetaldehyde; TAM: tryptamine; IAN: indole-3-acetonitrile; ILA: indole-3-lactic acid; TOL: indole-3-ethanol. Red lines: the IAA biosynthesis pathways in ZZ21 detected by genome-wide analysis; green lines: the IAA biosynthesis pathways in ZZ21 detected by metabolomic analysis; purple lines: the IAA biosynthesis pathways in ZZ21 identified by HPLC-MS.

We found that the IAM, IPyA, TAM and TSO pathways likely exist in ZZ21, as six possible functional genes in these pathways were detected via gene prediction and annotation (Figure S1; Table 1). The IAM pathway is the best-characterized pathway in bacteria [22,23]. In this two-step pathway, tryptophan is catalyzed to the intermediate IAM, which is then converted to IAA (Figure S1 and Figure 5) [4,8]. The main genes driving the IAM pathway are *iaaM* and *iaaH*, encoding tryptophan 2-monooxygenase and a specific IAM hydrolase/amidase, respectively [23,24,25]. We detected *iaaM*-homologous gene in the ZZ21 genome, as well as *aam* and *gatA*, which were predicted to encode amidases (Table 1). Amidase, which functions in the second step of the IAM pathway, has been purified from the *Arthrobacter* sp. strain J-1 [26]. However, *iaaM*, *aam* and *gatA* showed no significant changes in transcript levels in response to tryptophan and it is therefore unclear how much the IAM pathway contributes to IAA production in ZZ21. An early study in *P. syringae* pv. *savastanoi* demonstrated that IAA production dramatically increases in response to exogenously supplied tryptophan without significantly altering tryptophan monooxygenase activity [27] and the transcription of *iaaM* and *iaaH*. Similarly, Gaffney et al. demonstrated that the expression of these genes in the IAM pathway is continuous and does not require an exogenous supply of tryptophan [28]. However, tryptophan 2-monooxygenase encoded by *iaaM* is negatively regulated by both IAM and IAA [29]. Therefore, the genes involved in the IAM pathway are possibly insensitive to exogenous tryptophan but sensitive to feedback inhibition by its reaction products. Taken together, the existence of the IAM pathway in ZZ21 remains to be confirmed.

The IPyA pathway is thought to function in both plants and bacteria, including *Azospirillum*, *Enterobacter*, *Rhizobium*, and *Bradyrhizobium* [4]. In the first step of this pathway, tryptophan is converted to IPyA by an aminotransferase [30,31]. The conversion of IPyA to IAAld by indole-3-pyruvate decarboxylase (IPDC) (encoded by *ipdC*) is the rate-limiting step in this process [32,33,34,35]. IAAld is then oxidized to IAA by aldehyde dehydrogenase, mutase, or oxidase enzymes (Figure S1 and Figure 5) [36,37]. In *Bacillus amyloliquefaciens, dhaS* is thought to encode indole-3-acetaldehyde dehydrogenase [12,38]. The TAM pathway was identified in *Azospirillum* based on the conversion of exogenous TAM to IAA (Figure S1 and Figure 5) [39] and in *Bacillus cereus* based on the identification of tryptophan decarboxylase activity [40]. Nevertheless, the bacterial genes encoding related enzymes in this pathway must still be confirmed.

To date, the TSO pathway has only been demonstrated in *Pseudomonas fluorescens* CHA0. In this pathway, tryptophan is converted to IAAld and then oxidized to IAA (Figure S1 and Figure 5) [41]. In the ZZ21 genome, we identified *prr*, *puuC*, and *aldH*, which were predicted to encode aldehyde dehydrogenases involved in the final steps of the IPyA, TAM and TSO pathways. These three genes were found to encode aldehyde dehydrogenases in *Pseudomonas putida* KT2440 [42], *Escherichia coli* K-12 [43], and *Bifidobacterium bifidum* PRL2010 [44], respectively, but a recent study on vermicompost-borne bacteria suggested that *aldH* is likely not specifically involved in IAA biosynthesis [45]. While some aldehyde dehydrogenases show high specificity for a few substrates, others exhibit low specificity for a wide range of substrates [8]. Aldehyde dehydrogenase requires NAD+ or NADP+ as an electron acceptor to convert IAAld to IAA [46,47,48]. In the current study, quantitative reverse-transcription PCR (qRT-PCR) analysis indicated that *prr* and *aldH* were significantly upregulated in ZZ21 in response to tryptophan (Figure 1b). This result suggests that these two genes are thought to be involved in IAA biosynthesis in ZZ21 and *puuC* is not. However, neither the well-documented indolepyruvate decarboxylase *ipdC* gene in the IPyA pathway nor the tryptophan side chain oxidase/tryptophan transaminase genes in the TSO pathway were detected in ZZ21, nor were genes involved in the TAM pathway. Consequently, we were unable to determine which of these pathways is used by ZZ21 to synthesize IAA. The IAN pathway is not as well characterized in bacteria as it is in plants. The microbial enzymes responsible for the initial conversion of tryptophan to indole-3-acetaldoxime have not been detected in bacteria. Indole-3-acetaldoxime is subsequently converted to IAN by an indoleacetaldoxime dehydratase (encoded by *oxd* genes), which has been identified in several bacteria [49]. Finally, IAN is transformed into IAA by a nitrilase in a single step [50] or by a nitrile hydratase and an amidase in two steps (Figure S1 and Figure 5) [51]. In the current study, nitrile hydratase and nitrilase, which were reported in *Arthrobacter* [26,52,53], were not detected in the ZZ21 genome. To determine the pathway(s) used by ZZ21 to synthesize IAA, chemical methods might be needed to detect intermediates involved in various IAA biosynthesis pathways.

The presence of a particular IAA biosynthesis pathway in bacteria is often proposed based on the identification of the corresponding intermediates. IAM, IPyA, IAAld, TAM and IAN are the main intermediate products in tryptophan-dependent IAA biosynthesis [4,8,19]. According to our metabolomic analysis, only three indole compounds were detected among the metabolites of ZZ21, namely IAM, IPyA and ILA. The identification of IAM indicates that in *Arthrobacter pascens* ZZ21, IAA is synthesized by the IAM pathway. The intermediate IPyA and its enzymatic reduction product ILA were also found among the metabolites of ZZ21. Subsequently, using HPLC-MS, we demonstrated that ILA was present among the metabolites of ZZ21, and we also identified TOL, the enzymatic reduction product of IAAld. Taken together, these results suggest that the IAM and IPyA pathways are used by ZZ21 to synthesize IAA, even though the genes encoding aminotransferase and indole-3-pyruvate decarboxylase in the IPyA pathway were not detected in the ZZ21 genome (Figure S1), perhaps due to the limitations of genomic DNA fragmentation. Similarly, Mino reported that tryptophan is metabolized to IAA via IPyA and IAAld in *Arthrobacter* and that IAM also forms as a product of tryptophan oxidation in this genus [17]. In addition, a comparison of the relative intensities of intermediates involved in the IAM and IPyA pathways suggests that the IAM intermediate could accumulate much more easily than the intermediates IPyA and ILA in the IPyA pathway of ZZ21 (Figure 2). Interestingly, the IPyA pathway was much more sensitive to exogenous tryptophan than the IAM pathway in ZZ21 (Figure 1b and Figure 4), which may be due to the increased activity of the key enzyme encoded by *ipdC* and the increased expression of this gene after the addition of exogenous tryptophan. This hypothesis has been demonstrated in both *Azospirillum brasilense* Sp7 and *Pseudomonas putida* GR12-2 [35,54,55].

The failure to detect the TAM intermediate among the metabolites of ZZ21, combined with the results of the genomic analysis, strongly suggest that ZZ21 does not employ the TAM pathway to synthesize IAA. The TSO pathway has thus far only been found in *Pseudomonas fluorescens* CHA0 [41]. This observation, combined with the results of our genome-wide analysis, indicates that the TSO pathway is unlikely to exist in ZZ21. IAN was also not identified in ZZ21, which is consistent with the lack of genes encoding enzymes in the IAN pathway in the genome scanning experiment and suggests that IAA biosynthesis in *Arthrobacter* does not occur through the IAN pathway.

Since ZZ21 was able to produce IAA in the absence of an exogenous tryptophan supply (Figure 1a), we thought that the species might have the ability to synthesize tryptophan as an endogenous precursor or that a tryptophan-independent IAA biosynthesis pathway might function in ZZ21. The tryptophan-independent IAA biosynthesis pathway was first identified in *Arabidopsis thaliana*, as proposed by Normanly et al. [56,57]. The tryptophan-independent IAA biosynthesis pathway in bacteria was first demonstrated in *Azospirillum brasilense* using labeled tryptophan-feeding experiments [9]. However, no related enzymes in this pathway have thus far been identified. Thus, the existence of a tryptophan-independent pathway has not been confirmed and requires further study [58].

In conclusion, the results of this study indicate that the IAM and IPyA pathways function in tryptophan-dependent IAA biosynthesis in the plant-beneficial *Arthrobacter pascens* ZZ21 (Figure 5). Moreover, an uncharacterized tryptophan-independent pathway for IAA biosynthesis likely exists in *A. pascens* ZZ21.

## 4. Materials and Methods

### 4.1. Chemicals and Materials

l-tryptophan (Trp), indole-3-pyruvic acid (IPyA), indole-3-acetamide (IAM, 98%), and indole-3-ethanol (TOL, 97%) were purchased from Sigma–Aldrich (St. Louis, MO, USA). Indole-3-lactic acid (ILA, >98%) was purchased from Chemical Industry Co., Ltd. (Tokyo, Japan), and indole-3-acetic acid (IAA, >99%) was purchased from Aladdin (Shanghai, China). Methanol, pyridine, n-hexane, methoxylamine hydrochloride (97%), and *N*,*O*-Bis (trimethylsilyl) trifluoroacetamide (BSTFA) with 1% trimethylchlorosilane (TMCS) were purchased from CNW Technologies GmbH (Düsseldorf, Germany). l-2-chlorophenylalanine was purchased from Shanghai Hengchuang Bio-technology Co., Ltd. (Shanghai, China). All chemicals and solvents were analytical or High-performance liquid chromatography (HPLC) grade.

### 4.2. Bacterial Strain and Growth Conditions

*Arthrobacter pascens* strain ZZ21 (China General Microbiology Culture Collection Center, CGMCC accession no. 7325) was grown at 30 °C with shaking at 250 rpm for 48 h. Minimal liquid medium contained 5 g·L^−1^ glucose, 2 g·L^−1^ (NH_4_)_2_SO_4_, 0.5 g·L^−1^ NaH_2_PO_4,_ 0.5 g·L^−1^ K_2_HPO_4_, 0.2 g·L^−1^ MgSO_4_·7H_2_O, and 0.1 g·L^−1^ CaCl_2_·2H_2_O at pH 7.0. Luria-Bertani (LB) medium contained 10 g NaCl, 10 g peptone, and 5 g yeast extract. When required, 200 mg·L^−1^ l-tryptophan was added to the medium to stimulate IAA production.

### 4.3. Quantification of IAA Levels

IAA levels in the supernatants of *A. pascens* ZZ21 cultures were estimated spectrophotometrically according to the method of Gordon and Weber [59]. Each 2-mL aliquot of supernatant was combined with 2 mL Salkowski reagent (50 mL of 35% HClO_4_ + 1 mL of 0.5 M FeCl_3_) and incubated in darkness for 30 min at room temperature for color development. Absorbance was measured at 530 nm using a UV-VIS spectrophotometer (Uvmini-1240; Shimadzu, Japan). The IAA concentration was calculated by comparing the absorbance with a standard curve constructed using known concentrations of IAA.

### 4.4. Analysis of Genes Involved in IAA Production

Genomic DNA was extracted from *A. pascens* ZZ21, detected by 1% agarose gel electrophoresis, and separated into 400–500 bp fragments with a Covaris M220 Focused Ultrasonicator. An Illumina paired-end (PE) library (460 bp library) was constructed for sequencing analysis, and the entire bacterial genome was subjected to bioinformatics analysis. Illumina MiSeq sequencing technology was used to sequence the genome of strain ZZ21. Genomic rRNA and tRNA were predicted using Barrnap 0.4.2 and tRNAscan-SE v1.3.1 software, respectively. Genes were predicted using Glimmer 3.02 software. Deduced protein sequences of the predicted genes were Blastp-aligned (blast 2.2.28+) with the Nr, genes, string and GO databases to obtain annotation information for the predicted genes. The obtained predicted genes were blast-aligned (blastx/blastp 2.2.28+) with the KEGG database, and the specific biological pathway involved in the corresponding gene can be obtained according to the KEGG Orthology (KO) number obtained from the alignment. The genes were identified based on their annotated information, which predicted putative enzyme activities already known to function in IAA metabolism.

### 4.5. Quantitative Reverse-Transcription PCR Analysis of Genes Involved in IAA Biosynthesis

For functional gene validation assays, cDNA (complementary DNA) was directly extracted from *A. pascens* ZZ21 in minimal liquid medium with or without 200 mg·L^−1^ l-tryptophan using a CellAmp^TM^ Whole Transcriptome Amplification Kit (Real Time) (TaKaRa, Dalian, China). The resulting cDNA was diluted 1:100 in RNase-free (Ribonuclease-free) water and used as template DNA for quantitative reverse-transcription PCR (qRT-PCR) with a SYBR Premix Ex Taq Kit (TaKaRa, Dalian, China). The reactions were carried out on an ABI Step One Plus Real-Time PCR system under the following conditions: 3 min at 95 °C for denaturation, followed by 40 cycles of 10 s at 95 °C, 30 s at 55 °C, and 20 s at 72 °C. The primers used for RT-PCR are listed in Table 2. The 16s rDNA (ribosomal DNA) gene from ZZ21 served as an internal control. The qRT-PCR data were analyzed using the 2^−ΔΔ*C*t^ method.

### 4.6. Metabolomic Analysis of the IAA Biosynthesis Pathway in ZZ21

After 48 h of culture in minimal medium, the fermentation broth of *A. pascens* ZZ21 was collected by gravity, filtered through a 0.22 μm filter, lyophilized, and stored at −80 °C. The lyophilized metabolites were re-dissolved in 10 mL methanol-water (4:1, *v*/*v*), and 20 μL of internal standard (l-2-chloro-phenylalanine, 0.3 mg·mL^−1^ in methanol) was added to 1 mL of each sample solution. After centrifugation for 10 min (12,000 rpm, 4 °C), 200 μL of the supernatant was loaded into a glass vial and lyophilized in a freeze concentration centrifugal dryer (LNG-T98, Taicang, China). After adding 80 μL methoxyamine hydrochloride pyridine solution (15 mg·mL^−1^) to the vial, the sample was vortexed for 2 min before being subjected to an oximation reaction in a shaking incubator at 37 °C for 90 min. After incubation, the sample was combined with 80 μL of BSTFA (1% TMCS) derivatization reagent and 20 μL of n-hexane. After vortexing for 2 min, the sample was reacted at 70 °C for 60 min and allowed to stand for 30 min at room temperature prior to Gas Chromatography-mass Spectrometer (GC-MS) metabolomics analysis. Quality control samples (QC) were prepared by mixing equal volumes of extracts from all samples, each at the same volume as the sample.

An Agilent 7890B gas chromatography system coupled to an Agilent 5977A MSD system (Agilent Technologies Inc., Santa Clara, CA, USA) was used to analyze the derivatized samples. The derivatives were separated by a DB-5MS fused-silica capillary column (30 m × 0.25 mm × 0.25 μm, Agilent J & W Scientific, Folsom, CA, USA). Helium (>99.999%) was used as carrier gas, with a constant flow rate of 1 mL/min through the column. The injector temperature was maintained at 260 °C. An injection volume of 1 μL was used for split mode injection (split ratio 10:1). The initial oven temperature was 60 °C, which was ramped up to 125 °C at a rate of 8 °C/min, followed by ramping up to 210 °C at 5 °C/min, to 270 °C at 10 °C/min, and to 305 °C at 20 °C/min and was held at 305 °C for 5 min. The temperature of the MS quadrupole and ion source (electron impact) was set to 150 and 230 °C, respectively. The collision energy was 70 eV. Mass spectrometric data were acquired in full-scan mode (*m*/*z* 50–450), and the solvent delay time was set to 5 min. A QC sample was inserted after every 10 analytical samples to assess the reproducibility of the entire analysis.

ChemStation (version E.02.02.1431, Agilent, Santa Clara, CA, USA) was used to convert the file format of raw data from the apparatus to common data format (CDF). ChromaTOF (version 4.34, LECO, St. Joseph, MI, USA) was used to analyze the data. Metabolites were subjected to qualitative analysis by National Institute of Standards and Technology (NIST) and Fiehn database.

### 4.7. Identification of the Intermediates in the IAA Biosynthesis Pathway of ZZ21 by HPLC-MS

Indole derivatives were extracted from culture supernatants of *A. pascens* ZZ21 grown for 48 h in minimal medium supplied with 200 mg·L^−1^ l-tryptophan at 30 °C by centrifugation at 8000 rpm for 10 min. The culture supernatants were acidified to pH 2.5 using 1 M HCl and extracted twice using a double volume of ethyl acetate. A fraction of ethyl acetate was air dried and re-dissolved in one-tenth volume of methanol [60]. These methanolic extracts were subjected to HPLC-MS/MS analysis.

An Agilent Technologies 1200 Series RRLC system coupled with an Agilent 6410 triple-quadrupole mass spectrometer (Agilent Technologies Inc., Santa Clara, CA, USA) equipped with an electrospray ion source (ESI) and operated in positive ion mode was used for analysis. An Agilent ZORBAX Eclipse Plus C18 column (2.1 mm × 150 mm, 3.5 μm) (Agilent Technologies Inc., Santa Clara, CA, USA) with mobile phases A (methanol) and B (0.8% formic acid in water) (6:4, *v*/*v*) was used, with the column temperature set to 30 °C and a flow rate of 0.2 mL/min. The total run time was 10 min, and the injection volume was 0.2 μL. The positive ionization mode was used at a spray voltage of 4000 V. The nebulizer gas (N_2_) pressure was set to 30 psi with a flow rate of 10 L/min. The heated capillary temperature was 330 °C. Mass spectrometric scanning was performed from 50 to 500 mass. The molecular weight of IAA, IAM, ILA and TOL standards are 175.1, 174.2, 205.1 and 161.2, and after hydrogenation, the relative molecular masses of the four indole compounds are 176.1, 175.2, 206.1, and 162.2, respectively. The fragmentor voltage was 135 V for mass spectrometry analysis and 90 V for secondary ion mass spectrometry analysis.

### 4.8. Statistical Analyses

One-way analysis of variance (ANOVA) was performed, with a *p*-value of <0.05 considered to be statistically significant. All statistical analyses were conducted using SPSS 22.0.

## Figures and Tables

**Table 1:** Identity of proteins encoded by possible genes involved in IAA ^a^ biosynthesis in ZZ21.

IAA Biosynthesis Pathways	ZZ21 GID	Products and Entry Numbers in KEGG	NCBI Refseq or GenBank	Identity (%)
IAM	*iaaM* (orf0652)	tryptophan 2-monooxygenase (EC 1.13.12.3)	SLJ94339.1 (*Arthrobacter* sp. P2b)	88
	*aam* (orf3469)	amidase (EC 3.5.1.4)	WP_056629692.1 (*Arthrobacter* sp. Soil736)	90
	*gatA* (orf0389)	amidase (EC 3.5.1.4)	WP_087872787.1 (*Arthrobacter globiformis*)	81
IPyA/TAM/TSO	*prr* (orf1423)	aldehyde dehydrogenase (EC 1.2.1.3)	ELT45240.1 (*Arthrobacter nitrophenolicus*)	96
	*puuC* (orf2422)	aldehyde dehydrogenase (EC 1.2.1.3)	WP_003803492.1 (*Arthrobacter globiformis*)	94
	*aldH* (orf3473)	aldehyde dehydrogenase (EC 1.2.1.3)	WP_026555119.1 (*Arthrobacter* sp. 35W)	93

^a^ IAA: indole-3-acetic acid.

**Table 2:** Primers used in this study.

Primer Name	Sequence 5′–3′ ^a^
gatAF	CGAAACCACCATCCGCTACG
gatAR	TGGAACTGGCGAAGAAAGGC
iaaMF	CCTGGAAAGCCGCTGTGA
iaaMR	GCCGTAGAAGGTCTGCTCGTC
prrF	ACTTCGGTCCGCTGAACAAC
prrR	CATCTCCACGGCTTCCTGTT
puuCF	GCCGCAGCAATCTCAAGC
puuCR	CCAGCAACGCAGCGAAGT
aamF	GCAACATTGTCGGCTTCAGG
aamR	CGGACCGAAAGACCTGGG
aldHF	GCAACACGGTGGTCTGGAAG
aldHR	GCCCAACAGGAACGGAACC
16sF	GGTTGCGATACTGTGAGGTG
16sR	CTCCCACAAGGGTTAGGC

^a^ All DNA sequences are written in 5′ to 3′ orientation.

## Supplementary Materials

The following are available online at http://www.mdpi.com/1422-0067/19/2/443/s1.

## Abbreviations

IAA	Indole-3-aectic acid
IAM	Indole-3-acetamide
IPyA	Indole-3-pyruvic acid
IAN	Indole-3-acetonitrile
ILA	Indole-3-lactic acid
IAAld	Indole-3-acetaldehyde
TOL	Indole-3-ethanol
TSO	Tryptophan side-chain oxidase
TAM	Tryptamine
IPDC	Indole-3-pyruvate decarboxylase
Trp	Tryptophan
TIC	Total ion current
EIC	Extracted ion chromatography
HPLC-MS	High-performance liquid chromatography-mass spectrometry
qRT-PCR	Quantitative reverse-transcription PCR
MRM	Multiple reaction monitoring
KEGG	Kyoto Encyclopedia of Genes and Genomes
NCBI	National Center for Biotechnology Information
LB	Luria-Bertani
CK	Control check
KO	KEGG Orthology
NIST	National Institute of Standards and Technology
