# Supplementary material for: Indole-3-Acetic Acid Biosynthesis Pathways in the Plant-Beneficial Bacterium Arthrobacter pascens ZZ21
Source: Int J Mol Sci. 2018 Feb 1;19(2):443. doi: 10.3390/ijms19020443 (PMC5855665; doi:10.3390/ijms19020443)
Supplement: Supplementary file 1 [file ijms-19-00443-s001.pdf]

**Table S1** Sequencing data statistics

| Sample | Insert length (bp) | Raw data pair reads | Raw data total bases (bp) | Q20 (%) | Clean data pair reads | Cleam data single reads | Clean data total bases (bp) | Average coverage |
|---|---|---|---|---|---|---|---|---|
| ZZ21 | 460 | 3937952*2 | 1976851904 | 92.36 | 3899252*2 | 35987 | 1837619283 | 428X |

**Table S2** Indicator statistics of assembly results

| Sample | ZZ21 |
|---|---|
| No. of all scaffolds | 72 |
| No. of bases in all scaffolds (bp) | 4,615,764 |
| No. of large scaffolds (>1000 bp) | 39 |
| No of bases in large scaffolds (bp, >1000 bp) | 4,600,908 |
| Largest scaffold length (bp) | 635,947 |
| Scaffold N50 (bp) | 212,981 |
| Scaffold N90 (bp) | 89,228 |
| G+C content (%) | 65.72 |
| N rate (%) | 0.00024 |
| Total no. of contigs | 83 |
| No. of bases in all contigs (bp) | 4,615,753 |
| No. of large contigs (>1000 bp) | 44 |
| No of bases in large contigs (bp, >1000 bp) | 4,597,586 |
| Largest contig length (bp) | 635,947 |
| Contig N50 (bp) | 200,987 |
| Contig N90 (bp) | 82,521 |

**Table S3** Gene information statistics

| Sample | ZZ21 |
|---|---|
| Number of genes | 4321 |
| Total length of genes (bp) | 3,992,178 |
| Average length of a gene (bp) | 923.9 |
| Gene density (number/kb) | 0.94 |
| GC content in gene region (%) | 66.37 |
| Gene/Genome (%) | 86.49 |
| Intergenic region length (bp) | 623,586 |
| GC content in intergenic region (%) | 61.56 |
| Intergenic length/Genome (%) | 13.51 |

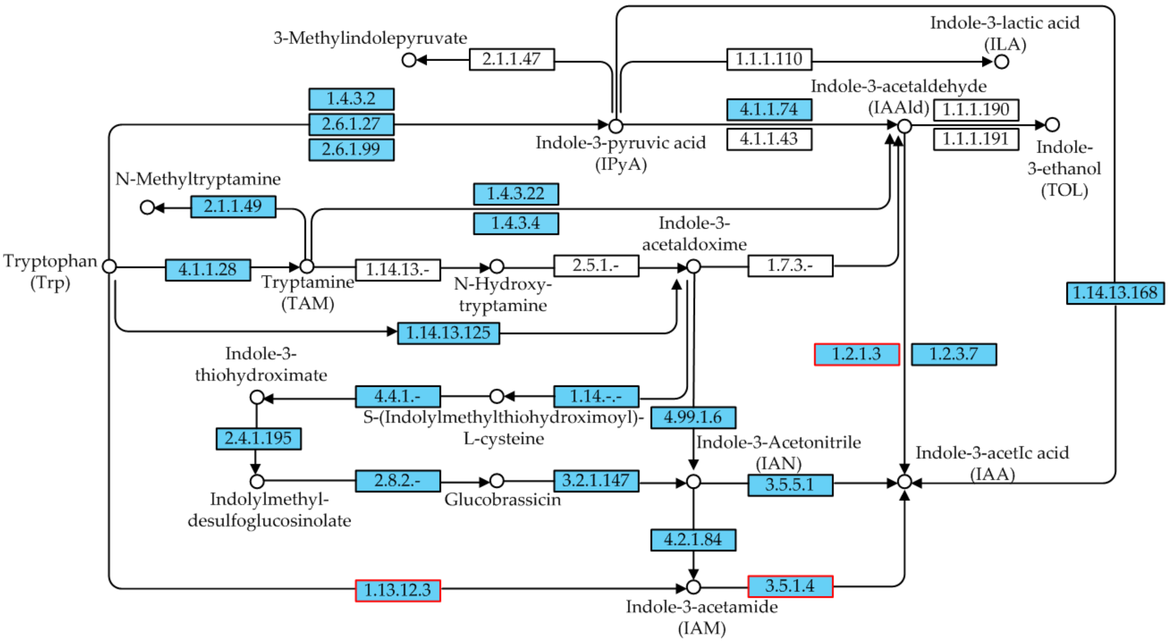


**Figure S1.** KEGG pathway involved in tryptophan metabolism (pathway entry number in KEGG: ko00380) and putative pathways involved in IAA biosynthesis in ZZ21. Circular nodes represent metabolic compounds, while rectangular nodes indicate gene products that are either classified as KEGG orthologues (blue) or not (white). A red border of a rectangular internode indicates that this enzyme is encoded by a gene in the *A. pascens* ZZ21 genome. Arrows represent enzymatic reactions or information transfer.

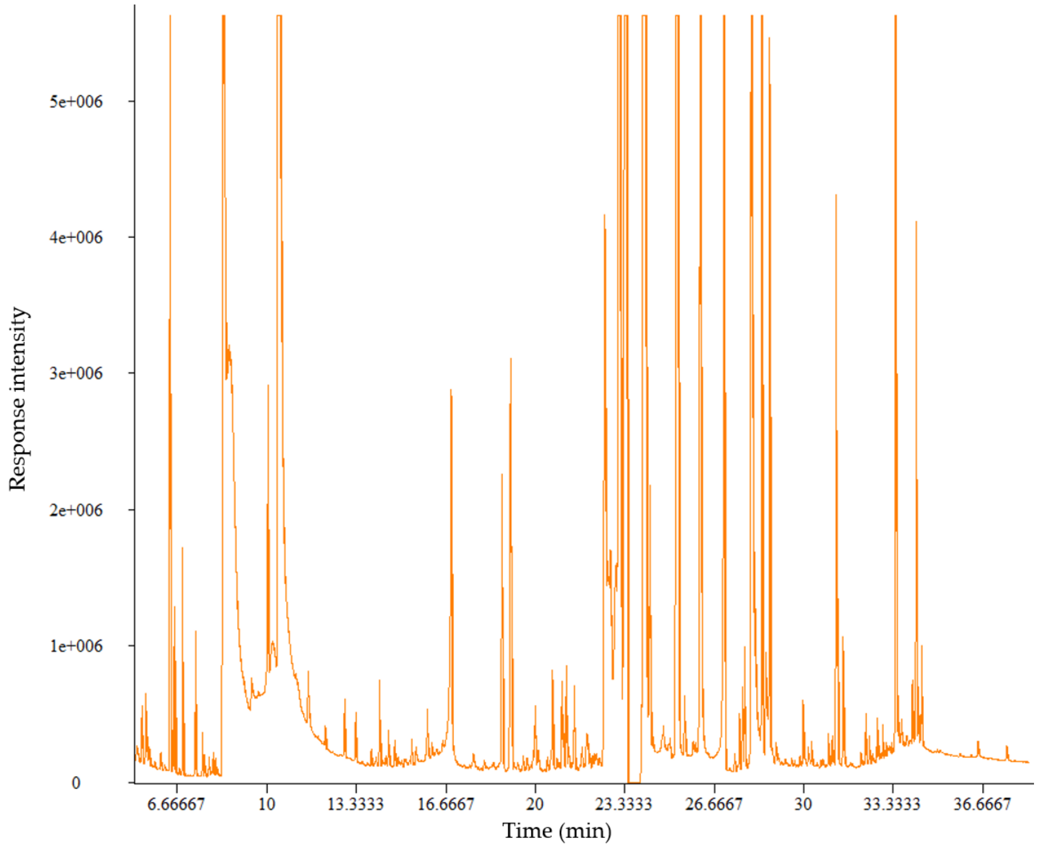


**Figure S2.** Total ion current (TIC) values of *A. pascens* ZZ21 metabolites. Mass spectrometric analysis was performed from 50 to 450 mass.
